# Immune Response to Dengue Virus Infection in Pediatric Patients in New Delhi, India—Association of Viremia, Inflammatory Mediators and Monocytes with Disease Severity

**DOI:** 10.1371/journal.pntd.0004497

**Published:** 2016-03-16

**Authors:** Mohit Singla, Meenakshi Kar, Tavpritesh Sethi, Sushil K. Kabra, Rakesh Lodha, Anmol Chandele, Guruprasad R. Medigeshi

**Affiliations:** 1 Department of Pediatrics, All India Institute of Medical Sciences, New Delhi, India; 2 Vaccine and Infectious Disease Research Center, Translational Health Science and Technology Institute, Faridabad, Haryana, India; 3 Institute of Genomics and Integrative Biology, New Delhi, India; 4 ICGEB-Emory Vaccine Center, ICGEB campus, New Delhi, India; U.S. Naval Medical Research Unit No. 2, INDONESIA

## Abstract

Dengue virus, a mosquito-borne flavivirus, is a causative agent for dengue infection, which manifests with symptoms ranging from mild fever to fatal dengue shock syndrome. The presence of four serotypes, against which immune cross-protection is short-lived and serotype cross-reactive antibodies that might enhance infection, pose a challenge to further investigate the role of virus and immune response in pathogenesis. We evaluated the viral and immunological factors that correlate with severe dengue disease in a cohort of pediatric dengue patients in New Delhi. Severe dengue disease was observed in both primary and secondary infections. Viral load had no association with disease severity but high viral load correlated with prolonged thrombocytopenia and delayed recovery. Severe dengue cases had low Th1 cytokines and a concurrent increase in the inflammatory mediators such as IL-6, IL-8 and IL-10. A transient increase in CD14^+^CD16^+^ intermediate monocytes was observed early in infection. Sorting of monocytes from dengue patient peripheral blood mononuclear cells revealed that it is the CD14+ cells, but not the CD16+ or the T or B cells, that were infected with dengue virus and were major producers of IL-10. Using the Boruta algorithm, reduced interferon-α levels and enhanced aforementioned pro-inflammatory cytokines were identified as some of the distinctive markers of severe dengue. Furthermore, the reduction in the levels of IL-8 and IL-10 were identified as the most significant markers of recovery from severe disease. Our results provide further insights into the immune response of children to primary and secondary dengue infection and help us to understand the complex interplay between the intrinsic factors in dengue pathogenesis.

## Author Summary

Dengue virus is a human pathogen that causes dengue fever, which can either resolve after mild fever or lead to severe dengue hemorrhagic fever/dengue shock syndrome. The role of dengue virus levels in the blood and the kinetics of infection and immune response that results in severe dengue disease in humans is not well characterized. In this study, we analyzed 97 children with varying degrees of dengue disease, and we show that the dengue virus quantity in blood does not show any significant association with severe disease. However, most severe dengue patients had lower levels of interferons and Th1 cytokines and increased levels of secreted factors such as IL-6, IL-8 and IL-10 that could potentially cause leakage in blood capillaries. Our results indicate that monocytes, which are infected with dengue virus in patients, could possibly play a major role in dengue pathogenesis. Furthermore, using computational analysis we identified association of some of the secreted factors with severe disease and also predicted the markers that could serve as indicators of recovery from severe dengue.

## Introduction

Dengue virus (DENV) infects close to 390 million people every year of which about 96 million have clinical manifestations that range from undifferentiated febrile illness to fatal shock syndrome [1]. There are four serotypes of DENV (DENV 1–4) and an initial infection with a single serotype (primary infection) has been proposed to cause less severe infections as compared to subsequent infections with a heterologous serotype (secondary infections). One of the mechanisms attributed to severe infections is antibody-dependent enhancement (ADE), wherein sub-neutralizing antibodies against primary infecting serotype bind, but do not neutralize the heterologous infecting serotype. These antibody-bound virus particles are endocytosed by Fcγ receptor-expressing cells, primarily monocytes/macrophages, enabling virus proliferation in these cell types [2]. A significant proportion of primary infections may also cause severe infections indicating a non-ADE mechanism of virus infection leading to severe disease [3]. Studies in dengue patients indicate that hyper-responsive immune system resulting in elevated levels of inflammatory cytokines as a cause of severe disease [4]. Much of the information on the pathogenesis of dengue and the role of antibodies and T cells therein has come from animal models lacking the interferon receptors [5]. However, understanding the kinetics of immune response to DENV infection in humans and to determine the role of correlates of protection or disease severity has been a challenge. Some of the characteristic clinical features of severe dengue disease in patients include thrombocytopenia, liver damage and disruption in the microvasculature leading to plasma leakage. There are contradictory findings from various groups regarding the secreted markers of disease progression and or severity and the role of dengue viremia in pathogenesis [6–11]. Most of these studies have been reported from tropical and sub-tropical regions of the world including South America, South- and South-East Asia. However, a cohort study to comprehensively investigate the kinetics of immune response in dengue patients and identifying the correlation between clinical parameters, plasma factors, viral load and the innate immune response from India has been lacking. India contributes to about 30% of global dengue infections and several areas of the sub-continent are hyper-endemic with multiple serotypes co-circulating throughout the year. In addition, drastic change in seasonal temperatures across the country is likely to give rise to varied patterns of dominance of region-specific serotypes. In the current study we established a pediatric dengue cohort in New Delhi and report the characterization of disease parameters and the correlation between the viral load, thrombocytopenia, plasma cytokine profile and severe disease in pediatric patients. Our findings may help in understanding the molecular mechanism of the interaction between the virus and the host immune response that culminate in severe dengue disease.

## Methods

### Ethics statement

The study was approved by the Institutional Ethics committees of all the three participating institutes (Ethics/THSTI/2011/2.1 dated 16 Nov, 2011; AIIMS: IEC/NP-338/2011 dated 17 Nov 2011; ICGEB/IEC/2011/01 dated 12 Nov 2011). Written informed consent for the study was taken from parents/guardians to collect blood samples at the time of admission and 48 h later. Patients were requested to come for a follow-up one month later for collection of convalescent samples.

### Screening and enrollment of dengue patients

Children aged between 4–14 years presenting to AIIMS with symptoms suggestive of dengue were screened using a “Dengue Day 1 Test” which is a rapid solid phase immuno-chromatographic test for the qualitative detection of dengue NS1 Antigen and differential detection of IgM and IgG antibodies to DENV in serum/plasma (J. Mitra and Co. Pvt. Ltd, New Delhi). Patients testing positive for either NS1 or IgM were informed and requested for consent for the study. Of 314 subjects tested, 153 (48.7%) had tested NS1/IgM positive on the rapid diagnostic test; of these parents of 97 patients consented for the participation of their child in the study (33 in 2012, 43 in 2013, 21 in 2014). We classified all patients at baseline bleeds and at the time of repeat bleeds (at least 48 h after baseline) based on the latest WHO grading into 3 groups—Severe Dengue (SD), Dengue with Warning signs (DW) and Dengue Illness (DI) [12]. At the time of enrolment, we graded 21 patients as DI, 30 as DW and 46 as SD. We admitted all patients with warning signs and severe dengue. The criteria for classifying children as having severe dengue were as follows: 34 patients (73.9%) had dengue shock syndrome (of which 5 were fatal and 2 more needed mechanical ventilation but survived); 8 patients (17.4%) had organ impairment, 6 patients had (13.0%) neurological manifestations (1 fatal); 2 (4.3%) had significant hepatic dysfunction; 3 (6.5%) had respiratory distress; 1 (2.2%) had severe haemorrhage.

The admitted patients were managed as per standard protocols of the admitting physician while the outpatients were followed up at alternate day intervals till recovery with the patient being free to visit earlier if necessary. A child was considered to have recovered clinically if the child was afebrile for more than 24 hours with subjective improvement in clinical condition, was hemodynamically stable. Patients were followed till 48 hours post-defervescence of fever OR subsidence of any other ongoing concerns as necessary. Overall six children (all had severe dengue at enrolment) died while 91 recovered. Patients were requested to come for a follow-up one month later for collection of convalescent samples. Complete blood counts were performed on automated COULTER COUNTER analyzer (Beckman Coulter) and serum chemistry was performed using COBAS analyzer (Roche Diagnostics).

### ELISA

Primary and secondary dengue was defined by performing capture ELISA for both IgM and IgG using Panbio Dengue IgM and IgG capture ELISA kits (Alere Product code 01PE10 and 01PE20) as per previous reports [12–15]. Primary dengue infections were those that did not show any detectable levels of serum IgM and IgG (seronegative) and those that had IgM > IgG by a ratio ≥ 1.2. Secondary dengue was defined as samples that had IgG only or IgG/IgM ≥ 1.2.

### Serotyping and viremia estimation

Total cellular RNA was isolated from 250 μl of whole blood obtained from dengue patients using Trizol LS reagent as per manufacturer’s protocol (Invitrogen). Dengue and Chikungunya detection was performed by FTD-43-Dengue-Chik multiplex RT-PCR kit as per manufacturer’s protocol (Fast-Track Diagnostics). Dengue serotyping PCRs were performed using FTD-44-64 single tube multiplex kit as per the manufacturers protocol using RNA isolated as described above. For a subset of samples, dengue serotyping was performed using nested PCR from patient RNA as described earlier [16]. 200 ng of RNA was used to estimate DENV viremia by Taqman one step RT-PCR as described previously except that human actin prime-probe mix (Applied Biosystems– 4326315E) was used as house-keeping gene for normalization [17].

### Multiplex cytokine/chemokine assays

25 μl of plasma from healthy controls/dengue patients/convalescent patients was used for assaying the cytokine levels using multiplex antibody bound-magnetic bead panels as per the manufacturer’s instructions (Merck-Millipore). Assays were performed in duplicates along with quality controls and absolute amount of each analyte was estimated by standard curve generated using known amount of analyte provided in the assay kit. Assay plates were read using Luminex-200 system and data was analyzed by Milliplex analysis software (Merck-Millipore) using five parametric logistic fit model.

### Peripheral blood mononuclear cells (PBMC) isolation, FACS and bulk sorting

Approximately 4–5 ml of blood sample was collected in Vacutainer CPT tubes (Becton Dickinson) and transported in a secondary container to the laboratory. Due to ethical restrictions for bleeding healthy children, 4–5 ml blood was collected from in-house healthy young adult volunteers as control samples for staining. The PBMC’s and plasma were separated according to the manufacturer’s instructions. The plasma was frozen at -80°C in aliquots and later used for ELISA’s and neutralization assays. The PBMC’s were washed extensively in 1% complete RPMI (RPMI containing 1% fetal calf serum (FCS), 1X Pen/Strep, 1X glutamine) and used immediately for analytical flow cytometry and cell sorting. For intracellular DENV staining, cells were first stained with surface receptor antibodies for 30 min on ice, washed extensively followed by fixation and permeabilization using Cytofix/Cytoperm (BD Bioscience) and followed by intracellular staining for 45–60 min on ice. Cells were washed extensively with 1X Perm wash (BD Bioscience) and 1X FACS Buffer (1X PBS containing 0.25% FCS) and immediately acquired on a BD FACS Canto II (BD Bioscience). For DENV staining, we used the commercially available 4G2 clone of the anti-flavivirus antibody (Merck-Millipore) that was conjugated to Alexafluor 647 using molecular probes conjugation kit (A20186) and other lymphocytes were stained with fluorochrome conjugated antibodies from Becton Dickinson (BD) CD3 PerCP (BD 347344), CD19 PE-CF594, (BD 562294), CD14-FITC (BD 555397), CD16-PE (BD 555407) and fixable viable dye efluor780 (ebiosciences 65-0865-14). For DENV staining, CD3+, CD19+, CD14+, CD16+ and CD14+CD16+ cells were gated after doublet discrimination. Frequency of DENV positive cells was plotted as mean fluorescence intensity (MFI). CD14 and CD16 analysis was performed after live cell gating, doublet discrimination and gating out the CD3+ and CD19+ cells.

For sorting experiments, PBMC's from patients were stained with CD3, CD14, CD16 and CD19 antibodies and these cell populations were bulk sorted on a BD FACS Aria III to a purity of 99% in 20% complete RPMI. The sorted populations were thoroughly washed and stored in Trizol at -80°C till RNA extraction was performed. Total RNA was isolated and cDNA was prepared using Verso cDNA synthesis kit (Thermo Scientific). IL-10 mRNA levels were estimated by RT-PCR using EvaGreen Supermix (Bio-Rad) (Forward Primer: 5’–TACGGCGCTGTCATCGATTT-3’ and Reverse primer: 5’- TAGAGTCGCCACCCTGATGT-3’) with GAPDH for normalization. Fold change was calculated by ΔΔCt method.

### Multivariate predictor analysis

In addition to the univariate tests of association/correlation, a multivariate feature selection algorithm, Boruta, based on Random Forests approach was taken to find the variables with maximum “predictive value” [18,19]. At the heart of this technique is an ensemble of decision trees called Random Forests and the importance of each mediator is calculated by simulating a “shadow dataset” [19,20]. R statistical platform was used to reproducibly clean up as well as analyze the data. Values below the detection range were replaced by an arbitrarily small and a large number respectively. Missing data were imputed by the median values of the respective variable. Boruta modeling for severity classification was done using the clinical definition of severity as the response variable and the complete set of data available at first bleed. These included demographic, clinical, hematological and molecular features. Specifically, Boruta has been shown to be the best performing algorithm giving consistent results [20]. The number of trees was optimized by inspecting for the stability of the results and was kept sufficiently large at 10,000 trees. Quality checks for clinical plausibility and dependencies of variables by serially eliminating the confirmed variables were performed. Inherent to the Random Forest algorithm is a calculation of the measure of importance of each variable for its predictive ability. The importance of the predictor is calculated by observing the change in error after a variable is randomly permuted, the intuition being that more decrease in prediction accuracy would be observed if a truly important variable was permuted and vice versa. However, the Random Forest algorithm fails to give a statistical measure of these importances. The Boruta algorithm performs a test for statistical significance of variable importances compared to a shadow dataset and outputs a distribution of Z-scores for each feature [19]. Only the features with Z-score statistically higher than the maximum achieved distribution for the shadow dataset were considered important. A similar analysis was carried out for discovering the minimal set of predictors for recovery. The predictor variables used were the absolute and relative differences between numerical variables obtained at the first bleed and the second bleed. In our analysis, Absolute Delta refers to the absolute difference between the pairs of values and the Relative Delta refers to this difference normalized by the value at first bleed. This was done because the patients might be in different stages of their disease/recovery at the time of the bleeds and hence quantifies the self-normalized difference for each patient.

### Statistical tests

All data were analysed by GraphPad Prism software. Statistical significance was assessed by Mann-Whitney test between the two groups. P values are indicated by: * = p<0.05, ** = p<0.005, *** = p<0.0005, **** = p<0.00005.

## Results

### Severe dengue cases in both primary and secondary infections

**Fig 1:**
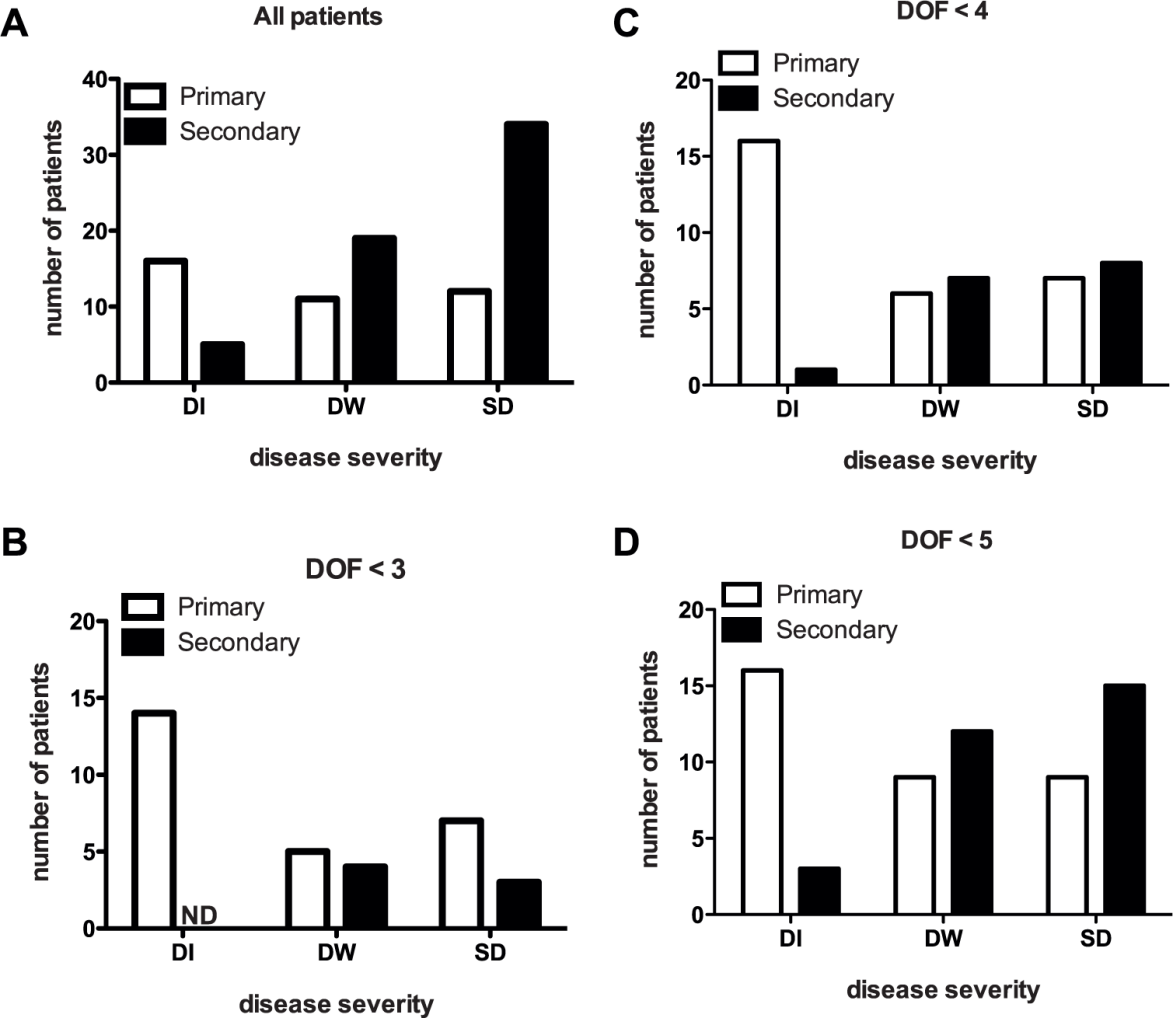
Severe dengue disease occurs in both primary and secondary infections. (A) Patients with primary and secondary dengue infections (N = 97) were stratified based on severity at the time of admission. DI–Dengue infection, DW- Dengue with Warning signs and SD–Severe Dengue. Patients with primary and secondary dengue infections with day of fever < 3 (B), <4 (C) or <5 (D) were stratified based on severity at the time of admission (N = 36, 45 and 64 respectively).

The clinical features of the 97 patients enrolled in the cohort are presented in Table 1. Day of fever (DOF) was significantly associated with severe disease. Fluid leak (based on clinical examination) was evident only in children with SD although this could be an underestimation as were unable to perform radiological examination on all patients; four of the SD cases displayed neurological symptoms such as encephalopathy and significantly more children with SD (60.9%) had hepatomegaly. Children with SD had lower platelet count at enrolment and their recorded nadir of platelet concentration was also lower compared to other groups. Hepatic enzymes- aspartate aminotransferase (AST) and alanine aminotransferase (ALT) were significantly higher in children with SD as compared to children with less severe condition. Serotyping by PCR confirmed 3 patients as DEN1 positive, 84 patients were DEN2 positive, 1 patient was DEN3 positive. Two patients were positive for dual serotypes (DEN 1 and 2 and DEN 2 and 4) (Table 2). Enrolled patients were confirmed to be negative for Chikungunya virus by real time PCR. Based on the IgG/IgM ratio, 58 of the 97 cases were classified as secondary infections (IgG>IgM), 11 patients had primary infections (IgM>IgG) and 28 were seronegative. Majority of the seronegative cases were from less than day 3 of fever, which is perhaps too early to detect IgM and these were classified as primary infections. Although the proportion of SD cases were higher in secondary infections (65.5%), about one-third of primary infections (31%) also caused severe disease (Fig 1A and Table 3). A large fraction of the enrolled patients had higher grades of disease severity due to the study site being a tertiary referral center and most of SD patients had day of fever (DOF) > 5. To further exclude recruitment bias in our observations, we determined the proportion of DI, DW and SD cases from primary and secondary infections in patients with DOF<3, DOF<4 and DOF<5 (Fig 1B and 1C). 79% of patients with DOF<3 had primary infection and this gradually decreased to 64% and 53% in DOF< 4 and DOF<5 groups respectively. Overall, 38% of SD cases from DOF<5 group were primary infections suggesting that both antibody-dependent and antibody-independent modes of infection may cause severe dengue in pediatric patients.

**Table 1:** Clinical and laboratory data on the day of admission of patients with dengue illness.

Variable^#^	Dengue Infection N = 21	Dengue with warning signs N = 30	Severe dengue N = 46	P value	Statistical test
Age in years; mean (SD)	9.5 (2.4)	8.9 (2.7)	8.5 (2.7)	0.40	ANOVA
Males: Females	11:10	15:15	25:21	0.93	Chi square
Day of fever	2.6 (2, 3.7)	4.5 (3, 5.6)	5.2 (3.9, 6.3)	0.0001	Kruskal Wallis
Rash; n (%)	6 (28.6%)	8 (26.7%)	10 (21.7%)	0.83	Fisher’s exact
Hepatomegaly; n (%)	4 (19.0%)	8 (26.7%)	28 (60.9%)	0.001	Fisher’s exact
Fluid leak*; n (%)	0	0	20 (47.8%)	<0.0001	Fisher’s exact
Encephalopathy; n (%)	0	0	4 (9.5%)	0.10	Fisher’s exact
Hemoglobin (g/dL) at enrolment; mean (SD)	11.9 (1.1)	12.3 (1.3)	12.0 (2.3)	0.63	ANOVA
Hematocrit at enrolment; %. Mean (SD)	37.1 (3.05)	37.3 (3.14)	37.5 (6.47)	0.95	ANOVA
Platelet count at enrolment; (X 10^3^/ μL)	94 (57, 182)	79 (38, 188)	43 (30, 79)	0.005	Kruskal Wallis
Maximum hematocrit during illness; %. Mean (SD)	38.8 (4.45)	40.1(4.74)	40.3(6.11)	0.54	Kruskal Wallis
Minimum platelet count during illness; (X 10^3^/ μL)	57 (35, 119)	44 (26, 93)	29 (16, 48)	0.0034	Kruskal Wallis
Viremia (genome equivalents/ml blood)	274000 (1160, 2065000)	26200 (147, 725000)	21400 (268, 1184000	0.6781	Kruskal Wallis
Aspartate aminotransferase; IU/L	37 (32, 131) N = 13	90 (54, 193) N = 20	130 (86, 297) N = 34	0.008	Kruskal Wallis
Alanine aminotransferase; IU/L	23 (16, 47) N = 13	36 (12, 56) N = 20	63 (27, 123) N = 34	0.024	Kruskal Wallis

^**#**^Values are median (Interquartile range) unless stated otherwise. SD- standard deviation

* clinical finding of fluid accumulation in serosal cavities was taken as evidence of fluid leak

**Table 2:** Dengue serotyping.

Serotype	No. of patients
DENV-1	3
DENV-2	84
DENV-3	1
DENV-4	0
Two serotypes	2
Negative/Undetermined*	7
Total	97

* These cases were confirmed to be dengue by other assays conducted to detect dengue antigen/antibody/virus.

**Table 3:** Disease severity of enrolled patients at the time of admission (bleed 1) and repeat bleeds at around 48 h post-admission (bleed 2) with primary and secondary dengue infections.

	*Bleed 1 (n = 97)*				*Bleed 2 (n = 53)*			
*Disease Severity*	DI	DW	SD	Total	DI	DW	SD	Total
**Primary**	16	11	12	39	7	6	2	15
**Secondary**	5	19	34	58	8	7	23	38

### Dengue viremia does not correlate with disease severity

**Fig 2:**
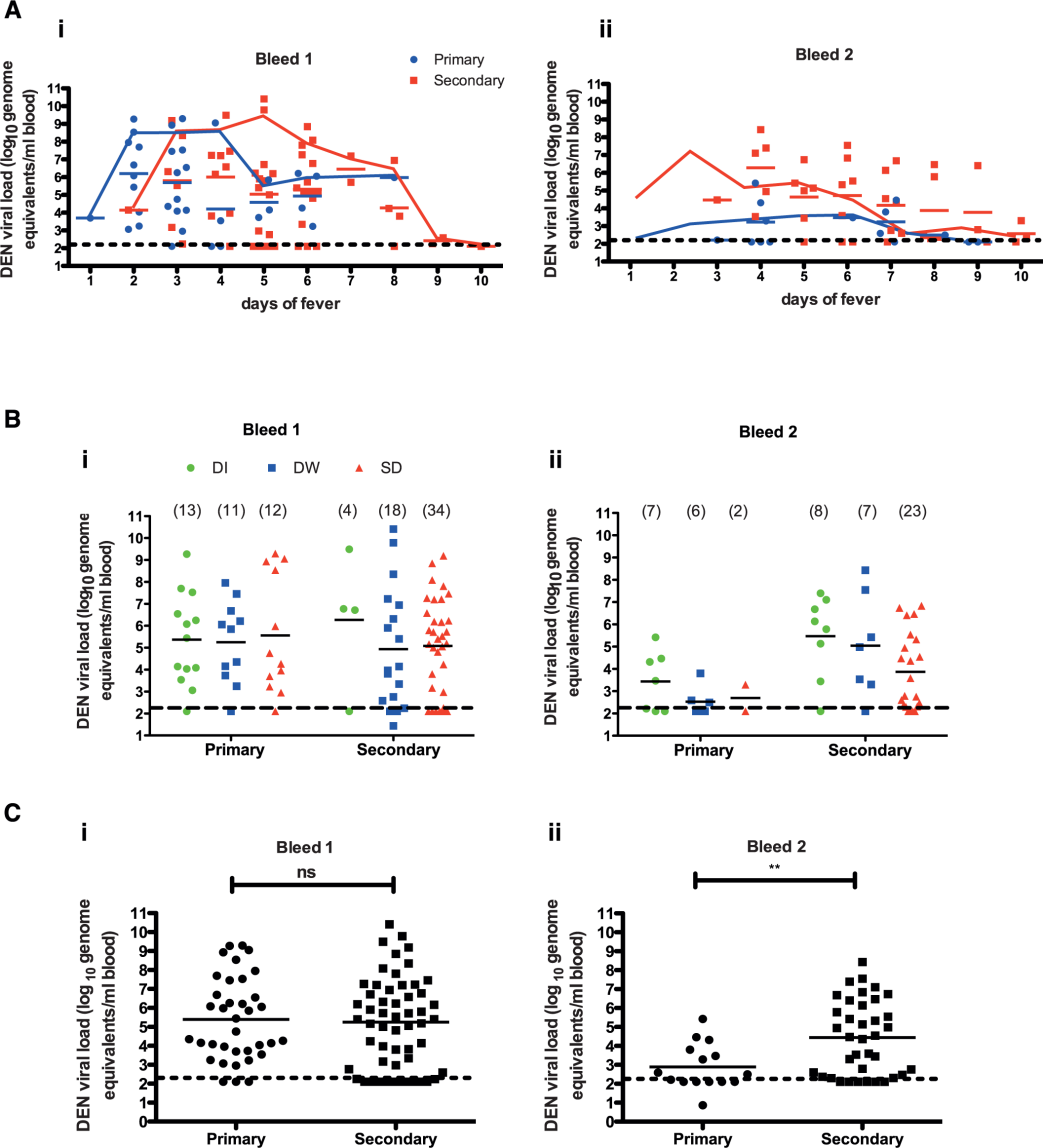
Dengue viremia does not correlate with disease severity. (A) Dengue viral RNA levels in patients classified as primary and secondary dengue infections in bleed 1 (i) and bleed 2 (ii) with respect to the day of fever. An overlapping connecting line graph shows median values of the respective scatter plot. (B) Relationship between dengue plasma viremia and disease severity at the time of admission (i) and 48 h post admission (bleed 2) (ii) in patients with primary and secondary dengue infection. (C) Overall comparison of dengue viremia in all primary and secondary infections in bleed 1 (i) and bleed 2 (ii). Geometric mean value of the scatter plot is shown in all figures. Statistical significance was determined by Mann-Whitney test. ** P = 0.0074.

There have been conflicting reports with respect to the role of viremia and infecting serotype in severe dengue disease [11,21–23]. Furthermore, antibody-dependent enhancement in secondary dengue infections has been proposed to augment viral replication leading to exaggerated immune response. To investigate these aspects, we determined dengue viral load in whole blood by real-time PCR. Dengue viremia was highest in patients with earlier DOF and showed a declining trend 6 days post-fever (Fig 2A(i)). Viremia was about ten-fold lower in repeat bleeds sampled 48 h after first bleed (Fig 2A(ii)). We found that both primary and secondary infections had comparable viremia in the first bleed (Fig 2AI and 2Ci) but patients with secondary infections had significantly higher viremia as compared to primary infections in the second bleed (Fig 2Aii and 2Cii). We found that dengue viremia was indistinguishable between patients with DI, DW or SD either in primary or in secondary infection but all the secondary infection cases in the repeat bleed had a significantly higher viremia as compared to primary infections despite showing clinical improvement from SD (Fig 2Bi and 2Bii) and 2Cii). These data suggests that DENV infects susceptible cells by both ADE-dependent and ADE-independent mechanisms, however, ADE may contribute to prolonged viremia observed in secondary infections most probably due to slower clearance of virus as reported earlier [24]. Next, we analysed viremia in patients with DI, DW or SD within day of fever 1–3, 4–6 or 7–10 groups. When we compared the distribution of disease severity among patients with respect to DOF, we observed that the proportion of SD cases increased from 31% to 52% and to 75% in patients with DOF 1–3, 4–6, 7–10 respectively. DI cases went down from 42% to 13% to 0% between DOF 1–3 to 4–6 to 7–10. DW cases remained almost constant across all patient groups (S1A Fig). We found no significant differences either in the first bleed or in the second bleed between different disease severities within the early, mid or late DOF groups (S1Bi and S1Bii Fig).

### Thrombocytopenia in patients with secondary infections and high viremia

**Fig 3:**
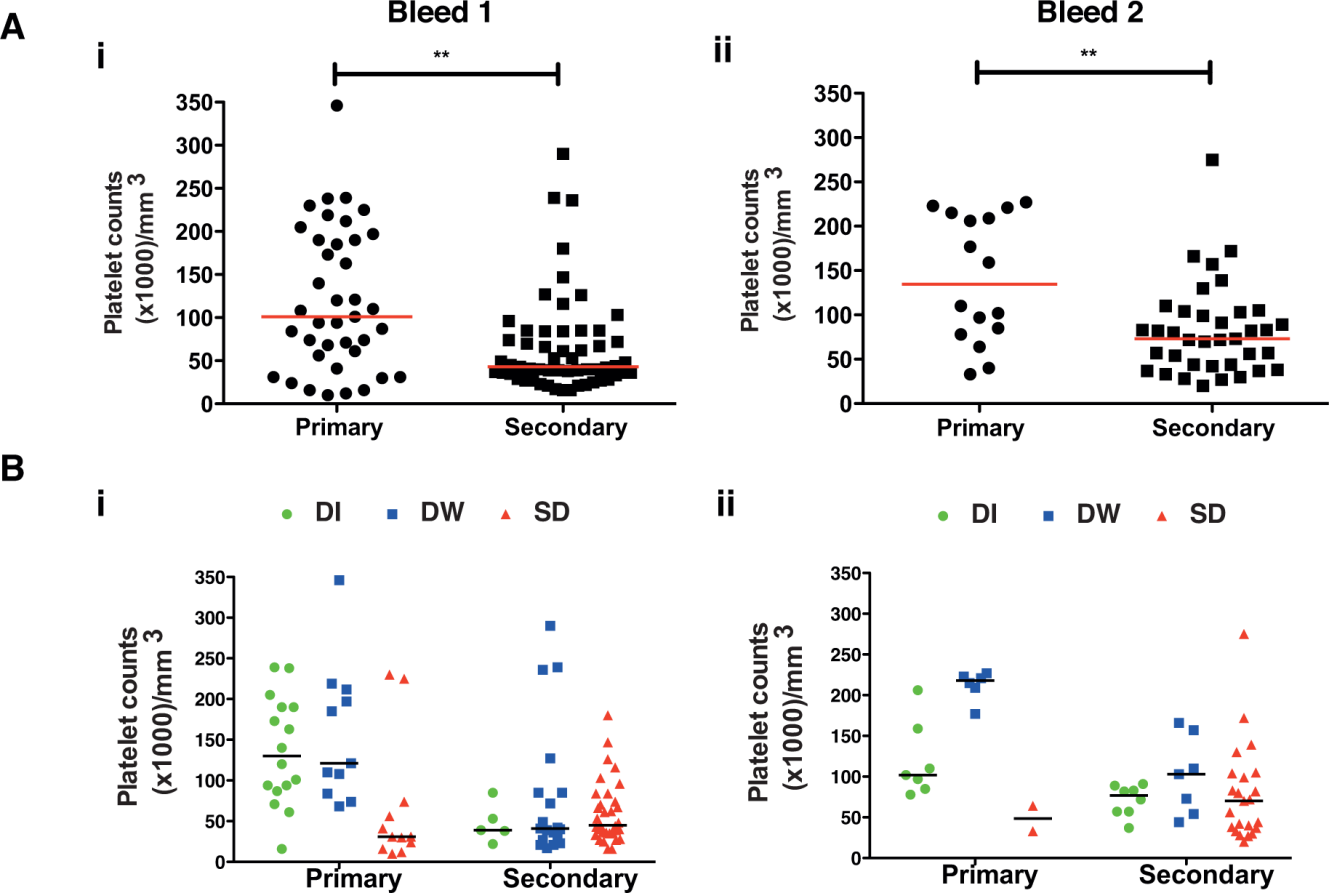
Relationship between platelet count and primary/secondary infections. (A) Platelet counts were measured at the time of admission, bleed 1 (i) or 48 h later, bleed 2 (ii) in patients with primary or secondary infections. (B) Platelet counts in bleed 1 (i) and bleed 2 (ii) of patients with primary or secondary infections categorized as per indicated disease severity. Median value is indicated. Statistical significance was determined by Mann-Whitney test. ** P< 0.005.

One of the characteristic features of severe dengue disease is thrombocytopenia but it is not clear if drop in platelet counts is a direct result of DENV infection or is a result of immune response or both. We next determined platelet numbers in patients at the time of enrollment and 48 h post-admission. Normal reference range for platelet numbers is 150,000–400,000 /mm^3^. Majority of the patients who came on day 3 of fever or later had drastically low platelet counts and, as expected, patients with SD had significantly lower platelet counts compared to DI or DW patients (S2A Fig and Table 1). Most of the patients with secondary infections showed significantly lower platelet counts both in bleed 1 and 2 corroborating that most secondary infections resulted in severe disease (Fig 3Ai and 3Aii). Further, platelet counts were marginally higher in DI and DW but lower in SD cases in primary infection whereas platelet counts were low in all three disease severities in secondary infection further indicating that severe disease in primary infection and secondary infections influence platelet homeostasis in dengue patients (Fig 3Bi and 3Bii). Although viremia had no correlation with disease severity, we investigated whether high viral load in blood has any association with platelet counts. To understand the link between viral replication and thrombocytopenia, we segregated samples into low viremia (<10^5^ DENV genome copies/ml blood) and high viremia (>10^7^ DENV genome copies/ml of blood). Platelet counts were significantly lower in patients with high viremia as compared to the ones with low viremia in the first bleed (S2Bi Fig). The viremia in majority of the patients who contributed second bleeds was below 10^5^ genome copies and the platelet counts had recovered in these patients (S2Bii Fig). These observations indicate that high viremia may contribute directly or indirectly to thrombocytopenia in a subset of SD patients. Some of the recent reports using animal models or *ex vivo* infection suggest that DENV may directly infect and replicate in megakaryocytes and platelets and may cause activation of platelets leading to apoptosis [25–27]. However, whether DENV infects platelets in humans and if this direct interaction between virus and platelets plays any role in thrombocytopenia observed in SD remains to be investigated.

### Cytokine kinetics and disease severity in dengue infections

Previous studies have associated cytokine storm or pro-inflammatory cytokine production in patients with severe dengue disease [28]. We measured the cytokine/chemokine quantities in patient plasma on the day of admission using multiplex magnetic bead assay for 14 analytes (IFN-α, IFN-γ, IL-6, IL-7, IL-8, IL-10, IL-12p40, IL-12p70, IL-17A, IP10, MCP-1, sCD40L, TNF-α and VEGF). There were significant differences in the levels of IFN-α, IFN-γ, IL-6, IL-7, IL-8, IL-10, IL-12p70, IP-10, MCP-1, sCD40L and VEGF in patients with DI, DW or SD infections (Table 4). IL-17A levels were below the level of detection in almost all the patients. IL-12p40, IP-10 and TNF-α levels did not show any significant difference between DI, DW and SD patients (Table 4).

**Table 4:** Quantitation of inflammatory mediators in dengue patients with different disease severities.

Analyte*	DI	DW	SD	P value^#^
IFN-α	38.7(15.6, 74.1)	12.8 (1.9, 38.4)	10.8 (5.6, 23.2)	0.0079
IFN-γ	9.4 (2.8, 35.2)	6.2 (1.9, 11.0)	3.8 (2.0, 7.5)	0.046
IL-6	1.6 (1.6, 2.4)	1.6 (1.6, 3.3)	13.2 (1.6, 25.4)	< 0.0001
IL-7	4.8 (2.8, 20.1)	3.6 (1.6, 9.0)	1.6 (1.6, 4.1)	< 0.0001
IL-8	12.0 (6.5, 19.5)	9.1 (6.4, 25.0)	29.5 (15.2, 73.3)	< 0.0001
IL-10	24.3 (13.1, 125.5)	86.2 (23.4, 206.8)	109.0 (46.0, 322.5)	0.0087
IL-12p40	1.6 (1.6, 10.3)	1.8 (1.6, 15.3)	3.3 (1.6, 7.1)	0.8289
IL-12p70	4.3 (2.5, 6.3)	3.3 (1.6, 6.1)	2.2 (1.7, 3.2)	0.0103
IL-17A	1.9 (1.6, 3.1)	1.6 (1.6, 2.8)	1.6 (1.6, 1.6)	∞
IP-10	7943 (4603, 10000)	9164 (6115, 10000)	10000 (7971, 10000)	0.0645
MCP-1	648 (373, 1005)	456 (288, 917)	384 (257, 616)	0.0428
sCD40L	4397 (947, 9066)	1570 (865, 3684)	405 (200, 1715)	0.0002
TNF-α	15.7 (13.1, 25.1)	17.1 (9.1, 26.3)	13.8 (10, 24.9)	0.7605
VEGF	44 (7.2, 91)	17.5 (1.6, 51.9)	38.4 (21, 62.7)	0.0468

* Values represent median with 25^th^ and 75^th^ quartiles.

# P value was determined by Kruskal-Wallis test

∞ IL17A levels were below the level of detection in almost all the samples

To further understand the kinetics of the immune response with respect to secretion of cytokines/chemokines and inflammatory mediators in dengue patients we segregated samples into three pools based on the DOF. We found two kinetic patterns of inflammatory mediator production in dengue patients namely: i) analytes that were elevated in patients with DOF 1–3 but showed a decline thereafter in patients with DOF 4–6 and 7–10 (IFN-α, IFN-γ, IL-7, IL-12p70, MCP-1) (S3A, S3B, S3D, S3H and S3J Fig); ii) analytes that were low in early phases of infection but showed a significant increase at later DOF (IL-6, IL-8, IL-10, IL-12p40, IP-10 and VEGF) (S3C, S3E–S3G and S3I Fig and S3M Fig). TNF-α did not show any significant difference between various DOF or between different disease severities (S3L Fig). sCD40L levels, compared to convalescent samples, were low in patients with DOF 1–3 and further decreased at later stages of fever (S3K Fig).

**Fig 4:**
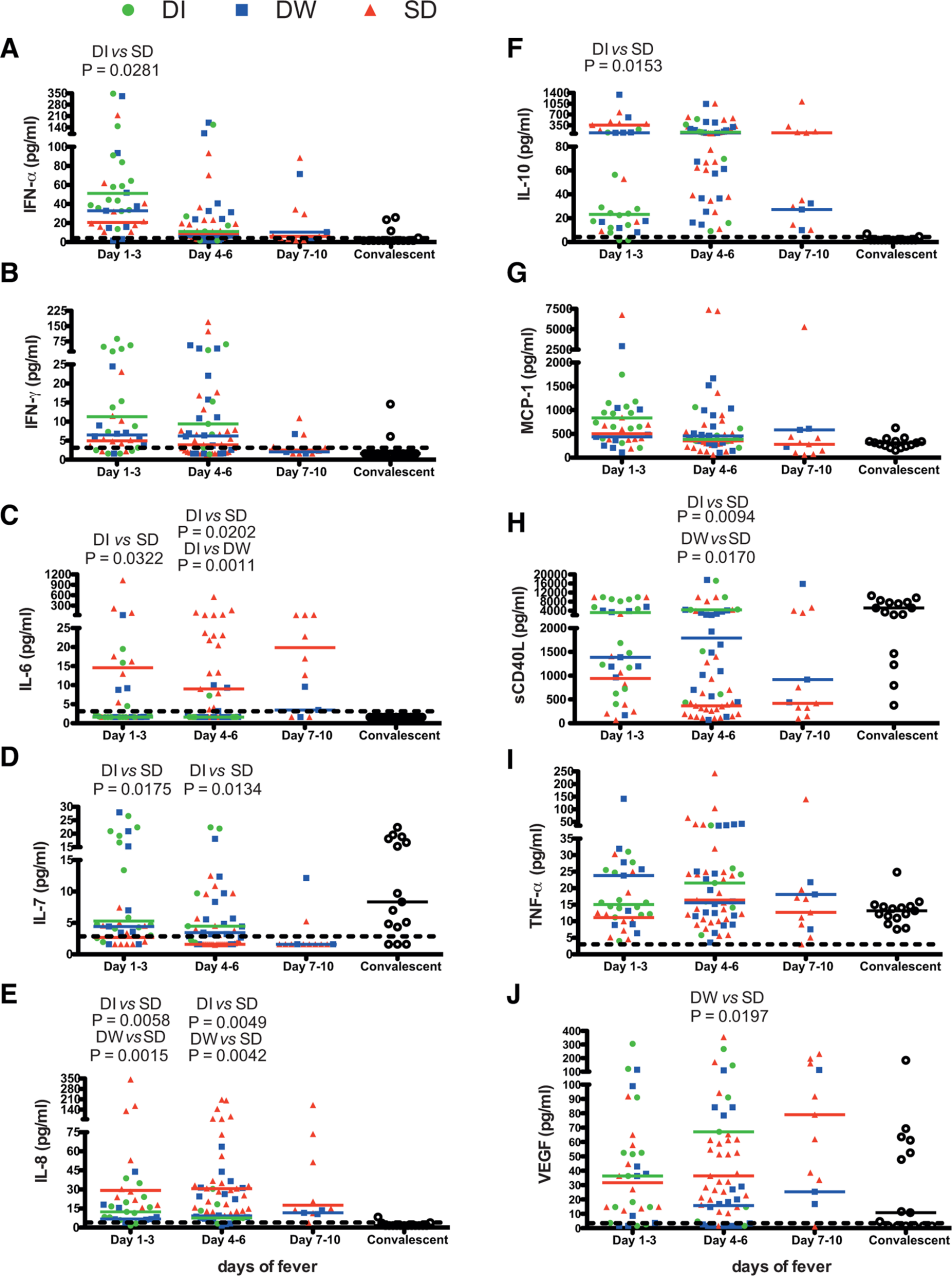
Cytokine profile in dengue patients on indicated days of fever and disease severity. Plasma levels of cytokines/chemokines/inflammatory mediators in dengue patients were measured by multiplex magnetic bead assays. (A) IFN-α (B) IFN-γ (C) IL-6, (D) IL-7, (E) IL-8, (F) IL-10 (G) MCP-1 (H) sCD40L, (I) TNF-α and (J) VEGF. Data are segregated into days of fever and disease severities within each group. Median value of cytokines in dengue illness (DI), dengue with warning signs (DW) and severe dengue (SD) is indicated by the respective color-coded bar. Dotted line represents the limit of detection. Statistical significance was determined by Mann-Whitney test.

We next analysed the above mentioned secreted factors from three severity groups of patients within each of the DOF groups (Fig 4A–4J). We found that IFN-α and IL-7 levels were significantly lower in SD cases as compared to DI (Fig 4A and 4D) whereas IL-6, IL-8 and IL-10 levels were significanlty higher in SD in DOF 1–3 group (Fig 4C, 4E and 4F). In DOF 4–6 group, IL-7 and sCD40L were significantly lower in SD patients compared to DI or DW cases (Fig 4D and 4H) while IL-6 and IL-8 showed the same increasing trend in SD patients (Fig 4C and 4E). IL-6, IL-10 and VEGF levels were elevated in patients with DOF 7–10 but this was statistically insignificant. Although both IFN-γ and MCP-1 showed a declining trend with increase in DOF, there was no significant difference within the DI, DW and SD cases in any of the three DOF groups (Fig 4B and 4G). These results indicate that low IFN-α, IL-7, sCD40L and high IL-6, IL-8, IL-10 and VEGF are defining features of severe dengue.

**Fig 5:**
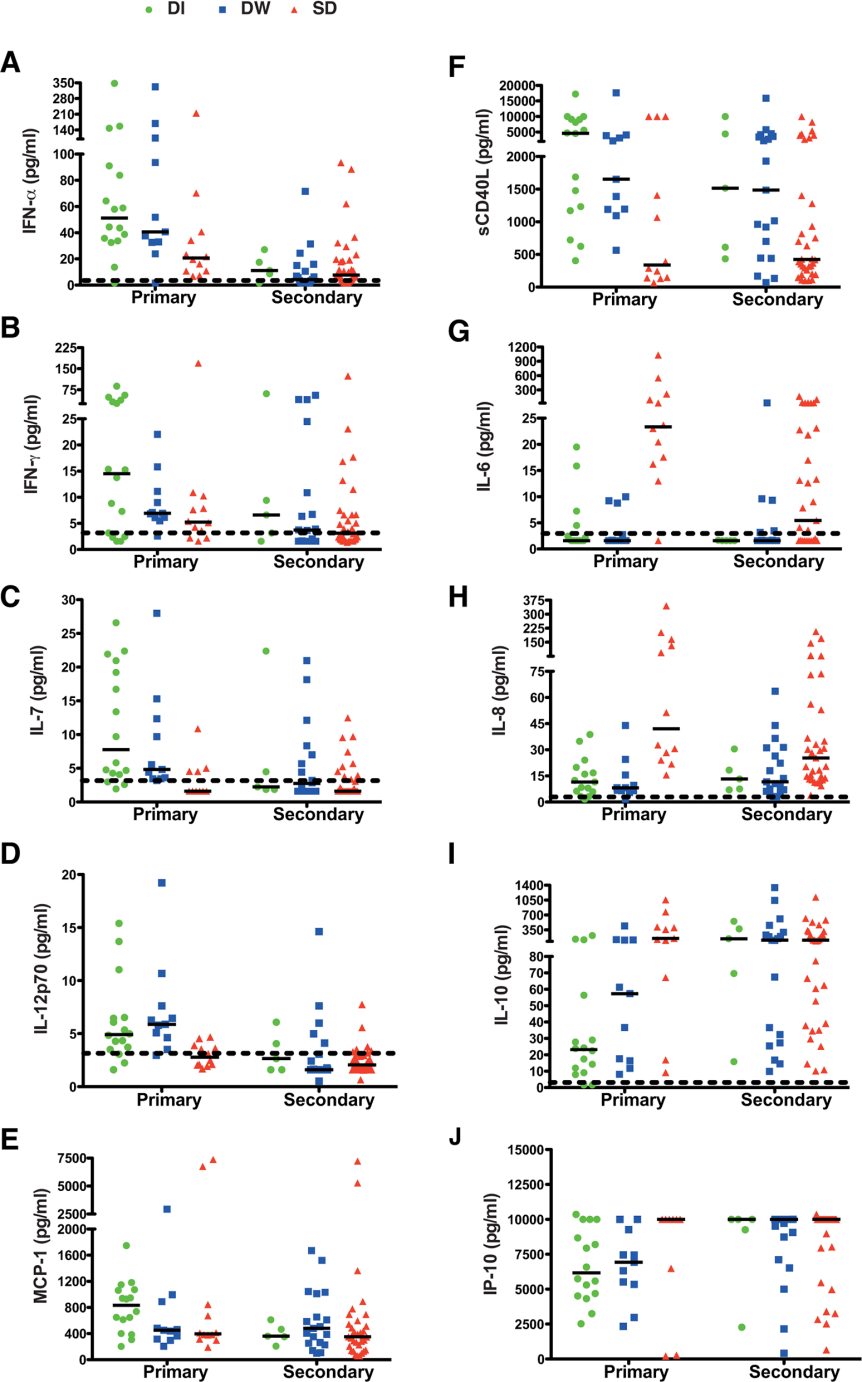
Cytokine profile in patients with primary and secondary infections Plasma cytokine/chemokine/inflammatory mediator levels in the plasma of primary and secondary dengue infection with indicated severities. (A) IFN-α (B) IFN-γ (C) IL-7, (D) IL-12p70, (E) MCP-1, (F) sCD40L (G) IL-6 (H) IL-8, (I) IL-10 and (J).IP-10. Median values are indicated by the bar. Dotted line represents the limit of detection.

40% of the enrolled patients in the cohort were primary dengue infections. Therefore, we next compared the cytokine secretion pattern in primary vs secondary dengue infections in patients with varying disease severities to determine if any of the secreted factors are different between these cases. The most important observation was that IFN-α, IFN-γ, IL-7, IL-12p70, MCP-1 and sCD40L were significantly reduced in secondary infections (S4A–S4F Fig). Interestingly, the levels of these analytes were reduced in all three disease severities in secondary infections whereas a graded reduction was observed between DI, DW and SD cases of primary infections (SD<DW<DI) (Fig 5A–5F). The levels of IFN-α, IFN-γ, IL-7, IL-12p70, MCP-1 and sCD40L in SD of primary cases were comparable with DI, DW and SD of secondary infections (Fig 5A–5F). Although no significant difference was observed between IL-6 and IL-8 levels in primary and secondary infections (S4I and S4J Fig), further segregation of disease severity within primary and secondary infection clearly showed higher levels of IL-6 and IL-8 only in SD cases of both primary and secondary infections (Fig 5G and 5H). Both IL-10 and IP-10 levels were higher in secondary infection as compared to primary infections (S4G and S4H Fig) showed a graded increase in DI, DW and SD infections in primary cases (SD>DW>DI) whereas DI, DW and SD patients with secondary infections had similar levels of IP-10 which was comparable with SD cases of primary infections (Fig 5I and 5J). TNF-α levels showed a marginal increase in secondary infections as compared to primary infections (S4K Fig) but the levels between DI, DW and SD was comparable (S5A Fig). VEGF levels were comparable between primary and secondary infection (S4L Fig) and between severties in primary infection however, in secondary infection, the levels were lower in DI and DW cases (S5B Fig). Altogether, these data suggest that severe dengue cases in either primary or secondary infections are characterized by similar changes in some of the key secreted factors measured in this study mainly, reduction in type I and II interferons, IL-7, IL-12p70 and sCD40L and increase in IL-6, IL-8 and IL-10.

### Association of viremia and inflammatory mediators in dengue infections

As high viral load was associated with a subset of patients with thrombocytopenia and severe disease, we next analysed correlation between viremia and the plasma levels of secreted factors described above. We found a siginificant, positive correlation between IFN-α, IL-12p40 levels and viremia. IL-7, sCD40L and TNF-α negatively correlated with viremia (Table 5). These data suggest that DENV replication, as expected, induces production of IFN-α and may also suppress IL-12 signalling pathway by inducing the inactive IL-12p40 which may compete with the active IL-12p70 for receptor binding. Our data also suggests a possible link between dengue virus infection leading to suppression of pro-survival cytokine IL-7 and T-cell homeostasis. Reduced sCD40L levels with high viral load provides further evidence of a link between viral load and platelet functions mediated by CD40-CD40L interactions.

**Table 5:** Correlation of viremia with secreted biochemical markers.

Analyte	r (95% CI)^#^	P value^#^
IFN-α	0.2637 (0.0536 to 0.4514)	**0.0120**
IFN-γ	0.0839 (-0.1316 to 0.2917)	0.4319
IL-6	0.1357 (-0.0797 to 0.3390)	0.2022
IL-7	-0.2609 (-0.4491 to -0. 0507)	**0.0130**
IL-8	0.1323 (-0.0831 to 0.3359)	0.2138
IL-10	0.0223 (-0.1917 to 0.2342)	0.8349
IL-12p40	0.2674 (0.0577 to 0.4546)	**0.0108**
IL-12p70	0.03701 (-0.1775 to 0.2481)	0.7291
IP-10	0.2046 (-0.0088 to 0.4003)	0.0530
MCP-1	0.0033 (-0.2099 to 0.2163)	0.9750
sCD40L	-0.2967 (-0.4795 to -0.0892)	**0.0045**
TNF-α	-0.2253 (-0.4183 to -0.0128)	**0.0328**
VEGF	-0.0467 (-0.2572 to 0.1680)	0.6620

# r and P value obtained by Spearman correlation analysis of all the samples with both viremia and cytokine measurements (n = 91).

### Distinct increase in CD14^+^CD16^+^ intermediate monocytes with dengue infection

**Fig 6:**
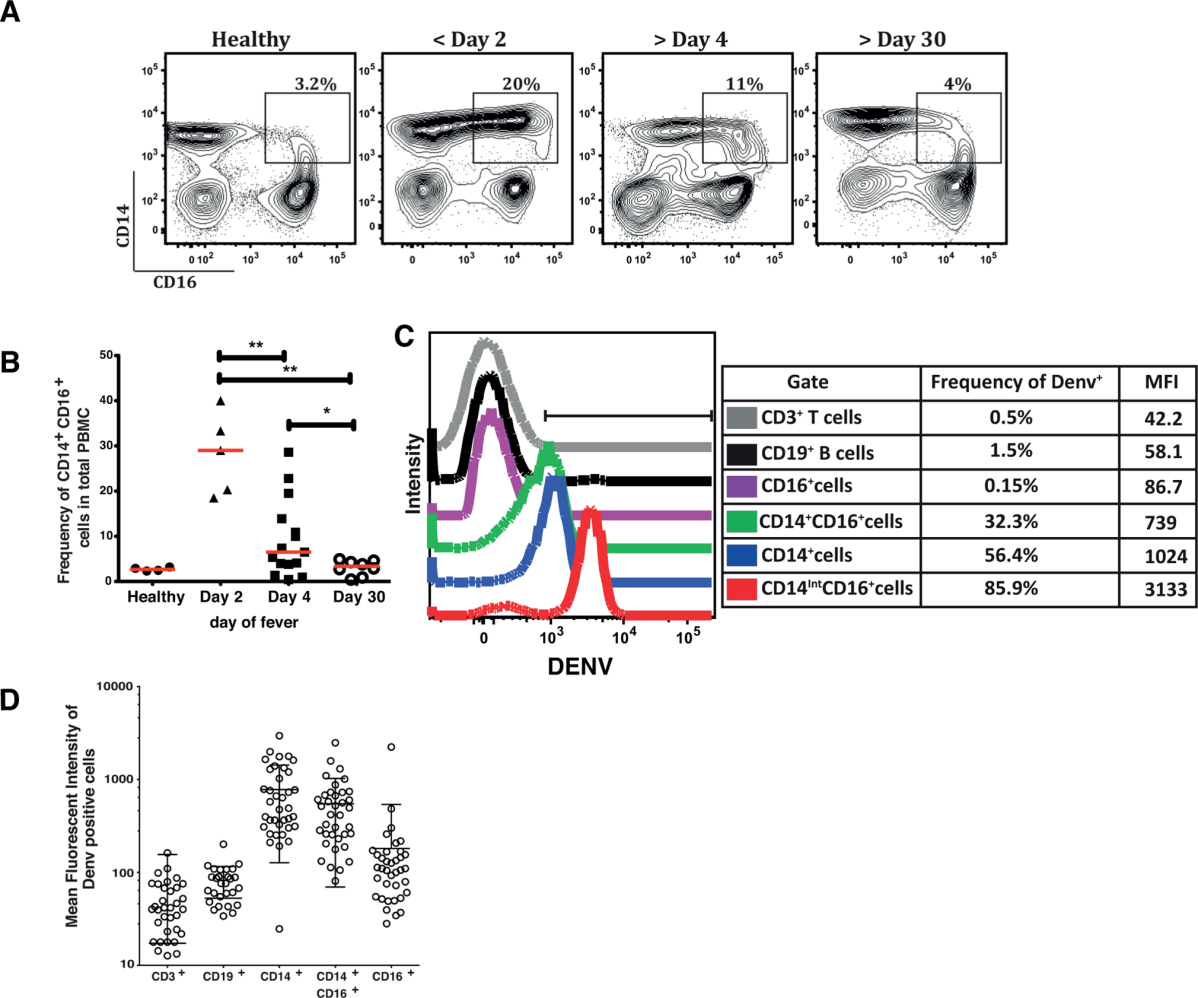
Transient increase in intermediate monocytes at early time points in DENV infection and monocyte infection with DENV. (A) Representative plots showing analytical multicolor flow cytometry of classical, non-classical and intermediate monocytes based on CD14 and CD16 expression *ex vivo* from PBMCs isolated from DENV-infected patients at indicated days of fever. (B) Cumulative frequencies of CD14+CD16+ intermediate monocytes as shown in scatter plot above. Unpaired, two-tailed t test was used for statistical analysis and P values were interpreted as * P<0.01 and ** P<0.001. (C) Ex vivo intracellular DENV staining was performed on total PBMCs stained with surface receptor antibodies for CD3, CD19, CD14, CD16 along with fixable viable dye to exclude dead cells. Representative histograms show intensity of DENV staining within each of the gated populations described. (D) Cumulative mean fluorescent intensities of DENV staining in the indicated cell populations (N = 31) is shown. Error bars represent Mean wth SD.

Monocytes have been shown to play a predominant role in the pathogenesis of dengue infection as they are permissive to dengue virus by both direct infection and by ADE. We next studied the magnitude of the monocyte response by multi-color flow cytometry in the peripheral blood samples of a subset of dengue patients (N = 31). Analysis of the CD14 and CD16 expressing cells revealed that there was an increase in the frequency of CD14^+^CD16^+^ intermediate monocytes very early (<day 3) after onset of fever. This increase was transient and started to decline by day 4 and was comparable to baseline in repeat bleeds from patients analyzed at day 30 post-acute dengue episode **(**Fig 6A and 6B**)**. The frequency of cell subsets were similar between healthy and convalescent samples (S6A and S6B Fig). DENV has been shown to infect T cells, B cells, monocytes and dendritic cells in *in vitro* models or in dengue patients [29–31]. We performed intracellular staining for DENV detection and we consistently observed DENV staining mostly in monocytes. Within the monocyte population, non-classical monocytes (CD14^-^CD16^+^) did not stain for DENV whereas the CD14^++^CD16^-^ classical monocytes and the CD14^+/Int^ CD16^+^ intermediate monocytes were positive for dengue antigen (Fig 6C and 6D)**.** The frequency of intermediate monocytes in total PBMCs reduced significantly by day 4 of illness (Fig 6C)**.** These data suggest that the frequency of intermediate monocytes increases very early after dengue virus infection and this cell population is markedly reduced upon disease progression indicating a potential role for these cells in severe dengue disease. To further confirm the contribution of monocytes in secreting inflammatory mediators in dengue infection we performed bulk sorting of PBMCs from two dengue patients and isolated CD3^+^, CD19^+^, CD14^+^ and CD16^+^ cells. Total RNA was prepared and IL-10 transcript levels were measured by qRT-PCR. As expected, we observed over 10-fold increase in IL-10 mRNA levels in dengue samples as compared to healthy control. CD14^+^ monocytes were the major contributors of IL-10 transcript (S7 Fig) further confirming the role of monocytes in the pathogenesis of dengue disease.

### Bioinformatics approach to identify markers of dengue disease severity and recovery

**Fig 7:**
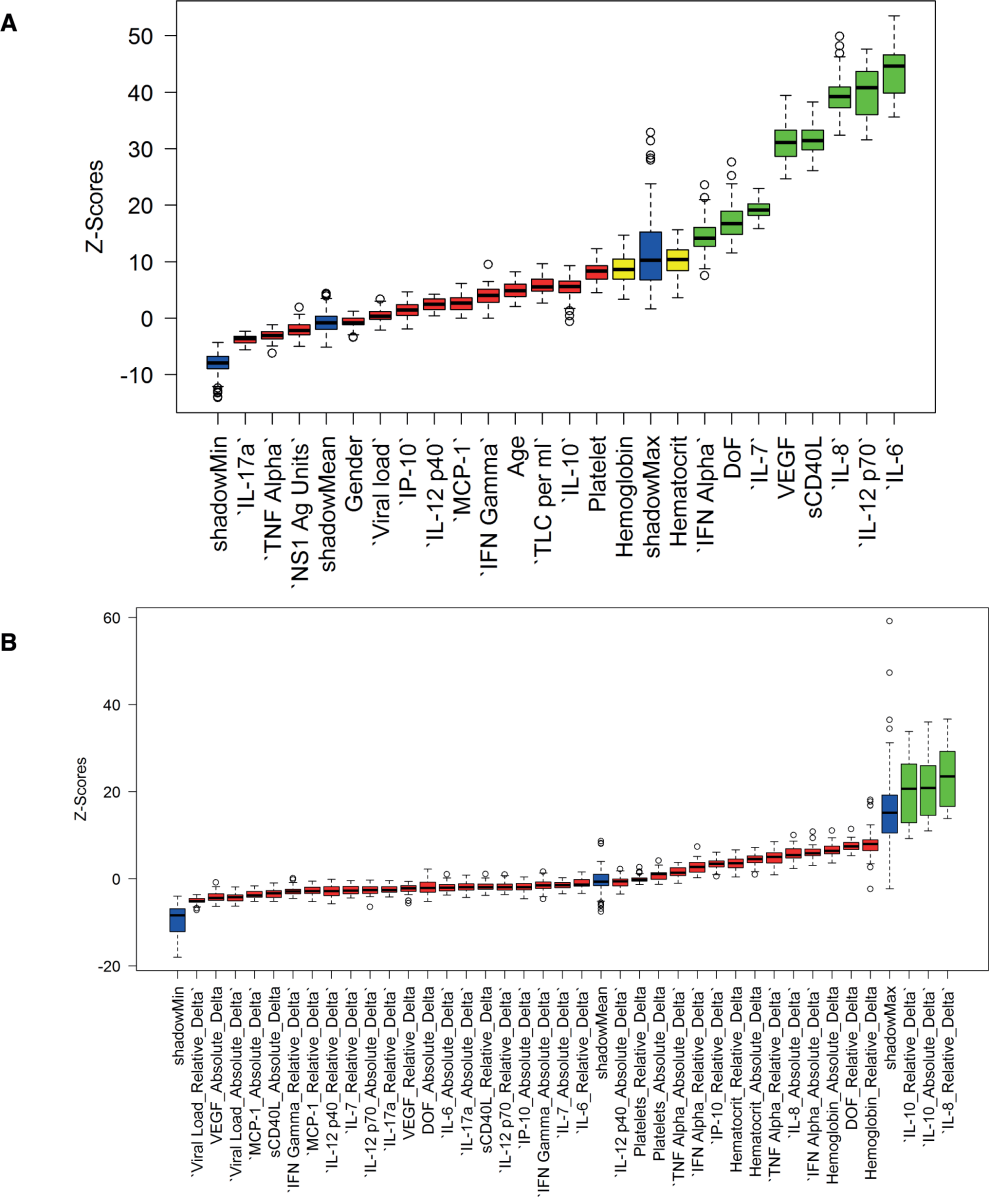
Multivariate selection of features of dengue severity and recovery. (A) The boxplots represent the distribution of variable importances after adjusting for the interactions among the variables. The minimal set of selected features (green) are the ones whose medians lie above the maximal possible median importance in the shadow data (see text for description). Eight parameters out of all available clinical and biochemical parameters were selected as important for prediction of the severity classes (SD, DW and DI). (B) Identifying markers of recovery from SD using the Boruta algorithm. All parameters from SD patients whose disease status improved clinically from first to second bleed were considered for analysis. Three delta parameters out of all the delta (absolute and relative changes across the first and second bleed, see text) were significantly important for prediction of recovery from SD and were consistent with findings in the univariate analysis.

Severe dengue has been proposed to be an outcome of exaggerated immune response and pathology resulting from the onslaught of inflammatory mediators such as IL-6, IL-8, IL-10 and TNF-α. However, there have been conflicting results regarding markers of dengue severity most likely due to the dynamic interplay between various factors of the immune system. Identifying markers of severe dengue disease needs to take into account the dependencies between the inflammatory mediators and clinical factors. We employed a machine-learning approach for variable selection, the Boruta algorithm, which learns the dependencies in the data from the data itself. The technique was carefully chosen because Random Forest-based techniques perform better in cases of the mixture of categorical and continuous predictors in the data and have desirable characteristics such as invariance to monotonic transformations, good performance in nonlinear datasets and the capability to auto-correct for dependencies between the variables. The advantage of this technique over standard variable importance obtained by random forests is in providing an estimate of the statistical significance of the importance values. Only the features with Z-score statistically higher than the maximum achieved distribution for the shadow dataset (shown in green in Fig 7A) were considered important. We found 8 factors namely, IFN-α, day of fever, IL-7, sCD40L, VEGF, IL-8, IL-12p70, and IL-6 whose Z-scores were above the threshold value of random set of importance to model the severity of the disease (Fig 7A). These data are in agreement with our observations and suggest that the decreased levels of IFN-α, IL-7, IL-12p70 and sCD40L in SD disease and increased levels of IL-8 and IL-6 play a predominant role as markers of disease severity in dengue infection. We further verified this by analysing the cytokine data in 6 of the SD patients (4 primary and 2 secondary, all DOF<5) who succumbed to infection. We found that in these patients dengue viremia was higher but not statistically significant compared to survivors of SD cases (S8A Fig). IL-6, IL-8, IL-10 and MCP-1 levels were significanlty higher in patients who died compared to the survivors (S8B–S8E Fig). There was no significant difference in TNF-α levels between these two group of patients (S8F Fig).

We next compared the factors that are predictive of the improvement in the clinical status of patients from SD. We hypothesized that the absolute and relative changes in quantitative markers (labeled as absolute delta and relative delta, with respect to the first bleed) would be important predictors of clinical improvement. As shown (Fig 7B), change in the levels of IL-8 and IL-10 were the only two statistically significant factors that coincided with the clinical recovery of patients. IL-10 seemed to be much more prominent as both delta and absolute delta were significant. This prediction was verified by comparing IL-8 and IL-10 levels in patients who had SD at the time of admission (first bleed) and had recovered clinically at the time of collection of second bleed (around 48 h post-admission). Most of these patients showed a significant decline in both IL-8 and IL-10 levels (S9A and S9B Fig) providing further validation for the relevance of prediction based on our computational approach.

## Discussion

India is hyperendemic to dengue and recent empirical estimates put the number of annual symptomatic infections to a staggering 6 million [32]. Despite the large annual dengue epidemics, there are no reports on the disease dynamics, virus isolates and circulation patterns, immune response and pathogenesis of dengue infection in Indian population. Here we present for the first time a comprehensive analysis of viral and immunological factors and its correlation with disease severity in pediatric dengue patients in New Delhi which is a hyperendemic region for dengue. About 40% of our study patients had primary infections and 31% of these patients had severe disease as against 65% of those with secondary infection. Dengue viremia remained at almost the same levels in patients with DOF 2–6, indicating a prolonged viral replication in children enrolled in our cohort. Additionally, significant number of patients with secondary infections took longer time to recover clinically and these patients had significantly high viral load as compared to primary infections suggesting that ADE may have contributed to higher viral replication in a subset of patients. Severe cases observed in primary infection suggests that ADE-independent mechanisms also contribute significantly to severe dengue which is in agreement with previous reports from other Southeast Asian countries [33,34]. Viral kinetics observed in our study is similar to some of the previous reports of DENV-2 infections from Thailand, French Polynesia and Vietnam [9,11,22,35,36]. Majority of the earlier studies with DENV-2 infections, either in adults or in children are reported as secondary infections. It has been suggested that DENV-2 primary infections are asymptomatic, and, therefore, higher proportion of secondary infections are DENV-2 positive [11,22,37]. 84 of the 97 patients in our cohort were DENV-2 positive and all patients showed clinical symptoms of the disease suggesting that primary DENV-2 infections can result in clinical presentations. The maximum viremia observed in our study is marginally higher than previous reports of DENV-1 infection in Chinese or Vietnamese adults or in secondary DENV-3 infection in Thai children [9,22,38]. Although the time of sampling could explain for this discrepancy, the differences in circulating virus strains as a contributing factor cannot be ruled out. Similar to our study, some of the other studies have failed to detect any significant difference in plasma viral RNA levels in mild vs severe dengue infections [33,35,36]. Vaughn et. al., reported that high viremia at early days of fever (DOF 3) correlates with severe disease at defervescence and that the duration of viremia had no correlation with disease severity. They also showed that primary infections (DENV1 and DENV3) had a prolonged viremia compared to secondary infections (all four serotypes) [11]. Our results are contradicting this observation as only two patients progressed from mild illness to severe dengue suggesting that high viremia at early DOF alone is not sufficient and other immunological factors with or without high viremia may determine progression to severe disease. Another explanation for our observation could be that plasma viremia does not totally reflect the localized viral replication taking place in tissue spaces which could eventually lead to vascular leakage due to inflammatory responses to this localized infection. We did not observe a significant difference in viremia between primary and secondary infections but secondary infections showed a prolonged viremia as compared to primary infections. Further investigations are underway to determine if the infecting virus strain in our cohort has an increased replication fitness or slower rate of clearance. Our study protocol allowed bleeding only once every 48 hours of illness. As many patients showed clinical improvement within 48 h and were discharged from the hospital, we were not able to collect second sample from all the patients to clearly define the peak viremia, day of illness and defervescence which is one of the limitations of the study.

The role of secreted factors, mainly cytokines and chemokines and molecular mechanism involved in dengue pathogenesis is yet to be proven conclusively as the events leading to severe dengue is likely to involve multiple factors [28,39]. Most of the previous studies have reported the association of some of the secreted factors with severe disease and its manifestations in secondary infections without correlations with viremia [10,40–43]. In agreement with some of these reports, we found no significant association between IFN-γ, TNF-α and severe dengue. We further show that reduced levels of type I interferon and IL-7 and elevated levels of IL-6, IL-8, IL-10 and VEGF strongly associated with severe disease both in primary and secondary infections. We show that severe dengue in primary infections exhibits the same profile and differential regulation of secreted factors analyzed in this study as secondary infections. By both univariate and multivariate analysis, we show that the most prominent features of severe dengue are lower type I and type II interferons, IL-7, sCD40L and increased production of IL-6, IL-8, IL-10 and VEGF. In addtion, our data indicates that inhibition of IL-12 signalling may also play a significant role in the manifestations of SD as IL-12 plays an important role in protective immune responses against pathogens by potentiating secretion of IFN-γ via a Th1 response and simultaneously suppressing IL-10 production [44]. We showed that significantly high levels of IL-6, IL-8 and IL-10 were present in patients who succumbed to dengue infection in our study. In addition, reduction in IL-8 and IL-10 was associated with improvement of clinical symptoms in severe dengue patients. Our results concur with previous findings where higher IL-10 levels were observed in patients with severe and/or secondary dengue infections [45–48]. However, other inflammatory cytokines such as IL-6 and IL-8 may also contribute independently to severe disease. IL-6, an acute phase reactant, was reported to be involved in suppression of IFN-β mRNA and induction of suppressor of cytokine signaling -1 (SOCS-1) and SOCS-3 expression in the *in vitro* model of ADE [49]. A previous report has shown high levels of IL-6, but not IL-12 or Foxp3+ (a marker of regulatory T-cells) cells, in dengue infected liver sections suggesting a critical role for IL-6 in dengue pathogenesis [50]. Previous studies have shown induction of IL-8 *in vitro* and also in severe dengue patients and IL-8 has been shown to induce vascular permeability *in vitro* suggesting that increased levels of IL-8 may contribute to SD [42,51,52].

We found a positive correlation between dengue viremia and IFN-α and IL-12p40 levels and negative correlation with IL-7, sCD40L and TNF-α. IL-7 provides the survival signal for peripheral T-cell pool [53] therefore, reduced IL-7 levels in severe DENV infection may have an effect on the survival and maturation of these cells. sCD40L is produced mainly by platelets upon activation, therefore, our data indicates a role for CD40-40L interaction in dengue infection which may lead to activation of platelets at early stages of infection. Interestingly, we found sCD40L levels to be highly correlative of platelet counts providing a useful surrogate marker for thrombocytopenia as platelets are the primary source of sCD40L in the circulation [54,55]. Whether sCD40L, which retains the receptor activation functions of CD40L, has any functional role in DENV infection warrants further investigation.

It is likely that the dynamic regulation of IL-10 levels at different stages of the disease involving different cell types determines the course of dengue disease. IL-10 is produced by different cell type in both acute and chronic phase of infection [56,57]. We show that CD14+ cells are the major producers of IL-10 and intermediate monocytes (CD14+16+) were the only cell type that was positive for DENV antigen. We recognize that DENV positivity by envelope antibody does not necessarily mean productive infection. We are currently assessing whether monocytes support active DENV replication in infected patients. Recently, DENV infection was shown to induce expansion of intermediate monocytes [58]. We show that induction of CD14+CD16+ cells occurs early in dengue infection and interestingly, patients with SD had reduced frequencies of CD14+16+ cells as was shown recently in Rhesus Macaques experimentally infected with DENV [58]. These results indicate that early immune response in DENV infection may be mediated by the infection-induced intermediate monocytes but further investigations are necessary to identify the role of different monocyte subsets and other cell types that contribute to immune responses leading to severe disease post-viral clearance.

Boruta algorithm has been shown to identify important features that are statistically significant in large data sets that may otherwise be masked in univariate analysis (VEGF as an example from our data). Using this approach, we identified some of the significant features as markers of severe dengue and also in the patients who showed clinical improvement from SD. Most of these factors were found to be important by univariate analysis thus verifying the validity and significance of *in silico* data. Our objective was to evaluate the core set of factors as it must be noted that the rejected variables might be independently important in a univariate analysis. In the presence of the significant set, these loose information and hence become un-important. For example, in the current data set, although platelet count is a major factor for labeling the clinical severity of the disease and hence was expected to be among the important variables, it was found that in the presence of the features listed above, platelet count did not remain important. This could be because the assays for the cytokines are more sensitive than the assays for platelet count or sCD40L levels may directly and more significantly reflect thrombocytopenia than the platelet counts thus highlighting the importance of more sensitive markers for severity classification of dengue. Further characterization of kinetics and cell source of IL-10 signaling at various stages of dengue disease may prove to be key to understanding the cause and effect of immune imbalance in SD. As reduced interferon levels seem to correlate with severe disease in both primary and secondary infections, therapies designed to transiently restore interferon levels to a threshold level required for recovery may prove to be useful in addition to antivirals and vaccine development.

## Supporting Information

S1 Fig Kinetics of dengue viremia in patients with varying disease severities. (A) Distribution of disease severity in indicated DOF groups is shown. (B) Dengue viral load as determined by real time PCR from whole blood samples of patients on the day of admission (bleed 1) and (B) 48 h post admission (bleed 2). Relationship between dengue virus genome copy numbers and disease severity at indicated days of fever. DI–Dengue infection, DW- Dengue with Warning signs and SD–Severe Dengue. Dotted line represents the limit of detection. Geometric mean of respective severities are indicated by color-coded bars. P values were estimated by Mann-Whitney test. Median values along with 25^th^ and 75^th^ percentiles are shown in the table for the indicated days of fever groups. P values were not significant between different DOF groups and between DI, DW and SD groups within each of the DOF groups.
(EPS)

S2 Fig Relationship between platelet counts, disease severity and viremia. (A) Platelet counts in patients with DI, DW or SD at the time of admission. (B) Platelet counts in patients with low or high plasma viremia in bleed 1 at the time of admission (i) and bleed 2 collected 48 h post-admission (ii). Median value is indicated. Statistical significance was determined by Mann-Whitney test.
(EPS)

S3 Fig Cytokine profile in dengue patients on indicated days of fever. (A-M) Cytokine levels in the plasma of convalescent controls and dengue patients were measured by multiplex magnetic bead assays. Median value of cytokines are indicated by the bar. Dotted line indicates the limit of detection. N.D.—Not detected. Statistical significance was determined by Mann-Whitney test. * P< 0.05, ** P< 0.01, *** P<0.0009, **** P<0.0001, ns–not significant.
(EPS)

S4 Fig Cytokine profile in dengue patients with primary or secondary infections. (A-L) Analysis of cytokine/chemokine/secreted factor levels in the plasma of dengue patients with primary and secondary infections. Median value is indicated. Dotted line indicates the limit of detection. Statistical significance was determined by Mann-Whitney test. * P< 0.05, ** P< 0.01, **** P<0.0001. ns- not significant.
(EPS)

S5 Fig TNF-alpha and VEGF profile in DI, DW or SD patients with primary or secondary infections. Analysis of TNF-α and VEGF levels in the plasma of dengue patients with primary and secondary infections and indicated severities. Median value is indicated.
(EPS)

S6 Fig Gating strategy and frequency of subsets in PBMCs of healthy adults and convalescent samples. (A) PBMCs were stained and analysed for CD14+, CD16+ and CD14+CD16+ subsets by gating CD3-CD19- population. (B) Frequency of each of the indicated cell subsets in healthy adults and convalescent samples in PBMCs is shown. Bars indicate median value.
(EPS)

S7 Fig Monocytes are the primary contributors of IL-10 levels in dengue infection. PBMCs from a DENV positive patient (dengue with warning signs) were processed by bulk sorting to isolate CD3+, CD14+, CD16+ and CD19+cells. Total RNA was isolated from sorted cells and IL-10 transcripts were measured by real time PCR. IL-10 mRNA levels normalized to GAPDH transcript. Error bars indicate standard error of experimental replicates. Graph is representative of one of the two sorting experiments from two patients. Error represents mean with standard error of the replicates.
(EPS)

S8 Fig Analysis of markers of severity in non-survivors. Comparison of DEN viremia (A), IL-6 (B), IL-8 (C), IL-10 (D), MCP-1 (E) and TNF-α (F) between severe dengue patients who succumbed to infection (n = 6) and the ones who survived (n = 40). Bars indicate geometric mean in (A) and median in (B-F). P values were estimated by Mann-Whitney test.
(EPS)

S9 Fig Indicators of recovery from severe dengue. Paired line graph indicating IL-8 (A) and IL-10 (B) plasma levels in DENV patients with severe disease at the time of admission (bleed 1) who showed clinical recovery two or three days later (bleed 2) (N = 17). The indicated P values were determined by Mann-Whitney test.
(EPS)
