# Correction: Immune Response to Dengue Virus Infection in Pediatric Patients in New Delhi, India—Association of Viremia, Inflammatory Mediators and Monocytes with Disease Severity

**DOI:** 10.1371/journal.pntd.0004642

**Published:** 2016-04-14

**Authors:** Mohit Singla, Meenakshi Kar, Tavpritesh Sethi, Sushil K. Kabra, Rakesh Lodha, Anmol Chandele, Guruprasad R. Medigeshi

There is an error in part A of Fig 2. The line graph in part ii of part A is shifted slightly to the left. Please see the corrected version here.

**Fig 2:**
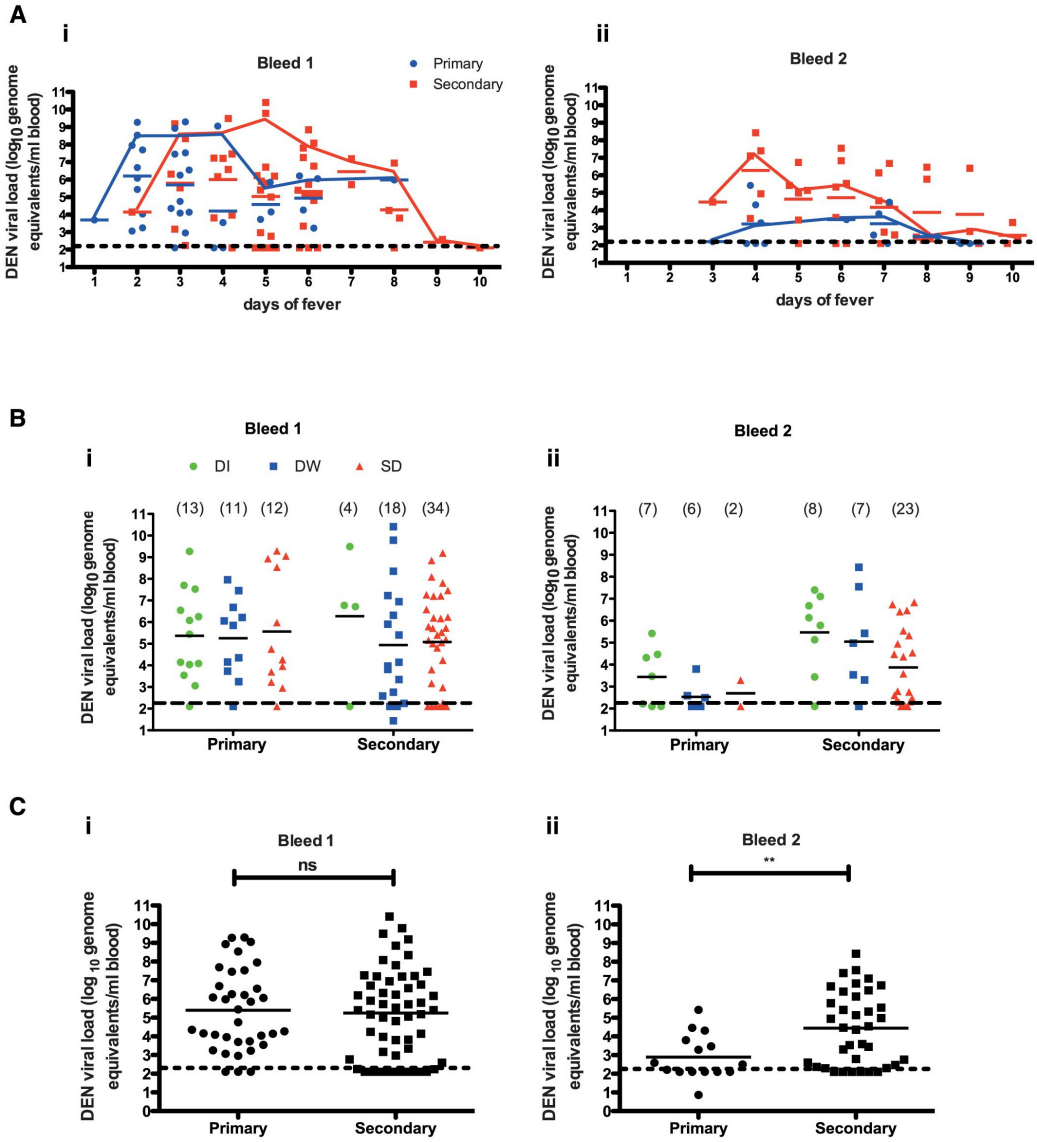
Dengue viremia does not correlate with disease severity. (A) Dengue viral RNA levels in patients classified as primary and secondary dengue infections in bleed 1 (i) and bleed 2 (ii) with respect to the day of fever. An overlapping connecting line graph shows median values of the respective scatter plot. (B) Relationship between dengue plasma viremia and disease severity at the time of admission (i) and 48 h post admission (bleed 2) (ii) in patients with primary and secondary dengue infection. (C) Overall comparison of dengue viremia in all primary and secondary infections in bleed 1 (i) and bleed 2 (ii). Geometric mean value of the scatter plot is shown in all figures. Statistical significance was determined by Mann-Whitney test. ** P = 0.0074.
